# Family Practitioners’ Advice about Taking Time Off Work for Lower Respiratory Tract Infections: A Prospective Study in Twelve European Primary Care Networks

**DOI:** 10.1371/journal.pone.0164779

**Published:** 2016-10-19

**Authors:** Maciek Godycki-Cwirko, Marek Nocun, Christopher C. Butler, Paul Little, Theo Verheij, Kerenza Hood, Nils Fleten, Anna Kowalczyk, Hasse Melbye

**Affiliations:** 1 Centre for Family and Community Medicine, Division of Public Health, Medical University of Lodz, Lodz, Poland; 2 Department of Toxicology and Carcinogenesis, Nofer Institute of Occupational Medicine, Lodz, Poland; 3 Nuffield Department of Primary Care Health Sciences, University of Oxford, Oxford, United Kingdom; 4 School of Medicine, University of Southampton, Southampton, United Kingdom; 5 University Medical Center Utrecht, Julius Center for Health, Sciences and Primary Care, Utrecht, The Netherlands; 6 Centre for Trials Research, Cardiff University, Cardiff, United Kingdom; 7 General Practice Research Unit, Department of Community Medicine, UiT the Arctic University of Norway, Tromsø, Norway; Ospedale Maggiore Policlinico, ITALY

## Abstract

**Background:**

Acute cough and lower respiratory tract infections (LRTIs) are one of the most important causes of lost working hours.

**Aim:**

to explore variation and predictors in family practitioners (FPs) advice to patients with LRTIs about taking time off work in different European countries.

**Methods:**

Prospective observational study in primary care networks in 12 countries, with multilevel mixed-effects binomial logistic regression.

**Results:**

324 FPs recruited 1616 employed adults who presented to primary care with LRTIs. The proportion of patients advised to take time off work varied from 7.6% in the Netherlands to 89.2% in Slovakia, and of these, 88.2% overall were advised to stay off work for seven days or less. None of Finnish or Dutch patients were advised to take more than 7 days off, in contrast to 35.5% of Polish and 27.0% of Slovak patients. The strongest predictors of FPs’ advice about time off work were: patient symptoms interfering with normal activities (OR 4.43; *P*<0.001), fever (2.49; *P*<0.001), patients feeling generally unwell (2.21; *P*<0.001), antibiotic prescribing (1.51; *P* = 0.025) and auscultation abnormality (1.50; *P* = 0.029). Advice to take time off was not associated with patient reported recovery.

**Conclusions:**

There is large variation in FPs’ advice given to patients with LRTIs in Europe about taking time off work, which is not explained by differences in patients’ reported illness duration, but might be explained by differences in regulations around certification and sick pay. Evidence based guidance for advising patients about taking time off work for this common condition is needed.

## Introduction

Acute cough and lower respiratory tract infections (LRTIs) are among the main causes of lost working hours in adults and the most common reason people take sick leave [1]. Respiratory tract infections (RTIs) are also among the most common diagnoses in primary care [2]. Such illnesses account for substantial part of family practitioners’ (FPs’) workload [3–5].

Although we have a good understanding of RTIs’ clinical presentation, diagnosing, treatment [6–9] and referrals [10, 11] there is still limited data on related physician behavior, such as advising patients on taking time off work. A common assumption is that adequate time to recuperate when unwell speeds recovery, helps prevent deterioration, and thus may facilitate earlier return to work [12, 13]. Those who consult frequently generally take more time off work [14]. Entitlement to be paid while off sick and associated isolation of unwell people at home may also reduce RTI’s transmission in the workplace [15, 16]. A Cochrane review found that implementing transmission barriers, including isolation, may be effective at containing respiratory viral epidemics [17], and may also reduce barriers to obtaining appropriate medical treatment [18]. Our searches did not identify evidence based guidelines for FPs about advising patients with LRTI to take time off work, but consensus-based guidelines have been developed in Sweden, recommending no sickness certification as a general rule for acute bronchitis and up to 14 days off work for pneumonia [19]. Substantial variation in regulations for sickness certification across the world has been found [12].

We aimed to explore the frequency and duration of FPs advice about taking time off work for LRTI and associated predictors in different European countries [20].

## Materials and Methods

The study was a part of European Union project GRACE-LRTI (Genomics to combat Resistance against Antibiotics in Community-acquired LRTIs in Europe).

### Study population

Study was performed in the winter season of 2006/2007, with participation of 324 FPs from primary care research networks in: Belgium (BE; 22 FPs), Finland (FI; 19 FPs), Germany (DE; 14 FPs), Hungary (HU; 11 FPs), Italy (IT; 12 FPs), Norway (NO; 35 FPs), Poland (PL; 21 FPs), Slovakia (SK; 23 FPs), Spain (ES; 41 FPs), Sweden (SE; 57 FPs), the Netherlands (NL; 28 FPs), the United Kingdom (UK; 41 FPs) who recruited patients during an initial consultation for an episode of acute cough. Eligible patients for this sub study were employed adults who presented to primary care physician with acute cough/LRTIs who were able to fill out study materials and gave written, informed consent.

### Data collection

FPs completed a case report form (CRF), rating symptoms as: “no problem”, “mild problem”, “moderate problem” or a “severe problem”. A total symptom severity score was calculated from the ratings of 14 individual symptoms/proxies (cough, phlegm production, breathlessness/shortness of breath, wheeze, coryza (blocked/runny nose), fever during this illness, chest pain, muscle aching, headache, disturbed sleep, feeling generally unwell, disturbance of normal activities, confusion, diarrhoea) and then scaled to range between 0 and 100.

Patients were asked to rate in a symptom diary the severity of 12 symptoms and effect on function on a seven-point scale (ranging from “normal/not affected’ (zero) to “as bad as it can be” (six)) each day until recovery (for 28 days). Symptom scores were calculated for each day (by summing the scores for each symptom). They were also asked to state the number of the day they felt recovered.

### Outcome assessment

Rates, variation and predictors in FPs’ advice to patients with LRTIs about taking time off work.

### Statistical analysis

Descriptive findings are presented as proportions (%), means and standard deviations inflated for clustering (FPs nested within the countries, mean±SD), or medians with interquartile range (IQR: 25%-75%). Predictors of FPs’ advising patients to take time off work were evaluated by multilevel mixed-effects binomial logistic regression with FPs and/or countries fitted as random effects, with the decision whether advise patients to take time off work as a dependent dichotomous variable. All modeling was performed in STATA statistical software.

## Results

### Patients’ demographics

Both CRFs and patients’ diaries with sufficient data were obtained from 79.1% (n = 2690) participants; 60.1% (n = 1616) were employed. Of 1616 employed patients, 79.3% worked full-time and 20.7% worked part-time; 51.1% (n = 826) were office workers, such as high level executive, major professional, administrative personnel, minor professionals, owners of small business. The mean age was 42.2±11.9 years, 63.5% (n = 1026) were females.

### Most common symptoms

The most common symptoms registered at presentation evaluated as a moderate or severe problem, were cough (85.6%), feeling unwell (50.2%), interference with normal activities (44.1%) and phlegm production (41.0%). The median number of symptoms presented by patients was 8 (IQR: 6–9) and the average symptom severity score was 31.0±14.1%. Detailed demographic and clinical characteristics of the working patients by country of residence are presented in Table 1.

**Table 1:** Demographic and clinical characteristic of working patients by country of residence (network). BE—Belgium, DE—Germany, ES—Spain, FI—Finland, HU—Hungary, IT—Italy, NL—Netherlands, NO—Norway, PL—Poland, SE—Sweden, SK—Slovakia, UK—United Kingdom. Data presented as mean±SD inflated for clustering or median (25%-75% IQR) or percentage related to the total number of cases in particular subgroups (%).

	BE (n = 86)	DE (n = 128)	ES (n = 197)	FI (n = 58)	HU (n = 210)	IT (n = 106)	NL (n = 79)	NO (n = 106)	PL (n = 124)	SE (n = 140)	SK (n = 195)	UK (n = 187)	All (n = 1616)
Age (years)	43.0±10.7	40.6±13.3	41.2±12.6	41.5±10.6	38.3±10.4	44.1±12.5	45.3±10.6	46.1±11.6	39.8±10.4	46.5±10.9	39.3±10.9	45.5±12.9	42.2±12.0
Males (%)	50.0	27.3	33.5	17.2	42.9	34.9	39.2	33.0	36.3	33.6	37.4	41.7	36.5
Females (%)	50.0	72.7	66.5	82.8	57.1	65.1	60.8	67.0	63.7	66.4	62.6	58.3	63.5
Part time work (%)	23.3	35.2	18.8	12.1	7.6	19.8	58.2	23.6	14.5	26.4	5.1	28.3	20.7
Full time work (%)	76.7	64.8	81.2	87.9	92.4	80.2	41.8	76.4	85.5	73.6	94.9	71.7	79.3
Office work (%)	58.1	46.1	50.8	32.8	25.2	71.7	74.7	39.6	59.7	42.9	64.6	57.8	51.1
Manual work (%)	41.9	53.9	49.2	67.2	74.8	28.3	25.3	60.4	40.3	57.1	35.4	42.2	48.9
Current smoker (%)	30.2	31.3	35.0	27.6	31.0	30.2	29.1	28.3	45.2	15.0	18.5	22.5	28.2
Feeling unwell before presentation (days)	4 (2–6.25)	4 (3–7)	4 (3–7)	5 (4–10.5)	2 (2–4)	5 (2–7.25)	8 (5–14)	6 (4–10)	4 (3–5)	8 (5.25–16)	4 (3–6)	5 (3–10)	4 (3–7)
Number of symptoms	8 (7–10)	8 (6–9)	7 (5–8)	9 (6–10)	7 (5–9)	6 (5–8)	8 (6–10)	8 (7–10)	8 (6–10)	9 (7–10)	8 (6–9)	9 (7–10)	8 (6–9)
Symptom severity score (%)	33.4±12.7	33.5±13.1	22.5±11.9	35.0±13.1	26.7±13.4	22.2±11.7	34.3±14.4	34.5±11.7	34.1±14.0	39.2±13.4	28.1±12.3	37.1±13.7	31.0±14.1
Discoloured phlegm (%)	41.9	41.4	33.5	62.1	41.4	35.8	48.1	57.5	31.5	57.9	43.6	57.2	45.0
^a^Comorbidity (%)	18.6	10.9	14.2	10.3	6.2	16.0	29.1	19.8	13.7	16.4	14.9	23.5	15.5
Auscultation abnormality (%)	40.7	29.7	24.4	43.1	96.2	57.5	40.5	48.1	50.0	31.4	62.6	40.1	49.2
Diagnosis of URTI (%)	43.0	3.9	48.2	10.3	11.9	33.0	46.8	17.9	38.7	11.4	42.1	19.8	27.4
Diagnosis of LRTI (%)	38.4	46.1	30.5	70.7	79.0	45.3	41.8	50.9	39.5	74.3	47.7	43.9	50.9
Antibiotics prescribed (%)	20.9	32.8	23.9	46.6	75.2	74.5	39.2	27.4	70.2	37.1	88.7	65.8	53.6
^b^Advised delay of antibiotic treatment (%)	22.2	2.4	10.6	11.1	13.3	16.5	3.2	10.3	11.5	9.6	4.6	34.1	13.4

^a^comorbidity (diabetes, or cardiovascular- or respiratory-related);

^b^related to number of patients prescribed with antibiotics.

### National networks variations in advising

**Fig 1:**
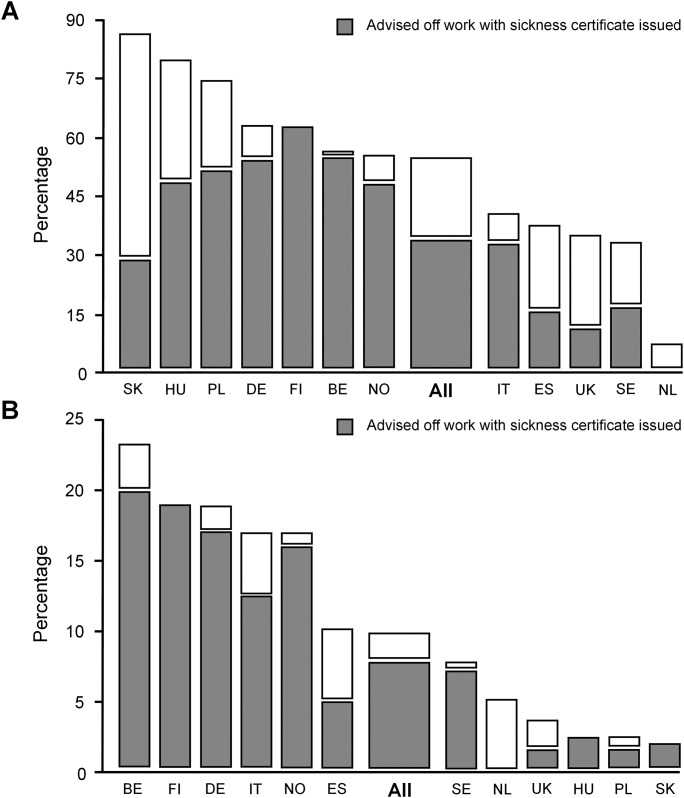
FPs’ advice to take time off work for LRTI. Panel A: FP’s advice to take time off work for LRTI by network—data presented as percentage of the employed population (full or part time) of those advised to take time off work by their FPs. Panel B: Proportion of employed patients reporting sickness certification as major reason for consulting their FPs by network—data presented as percentage of patients who indicated that sick certification was the main reason for consulting their FPs in relation to the group of working population. Insert grey bars represent the proportion of patients who received a formal sickness certificate.

**Fig 2:**
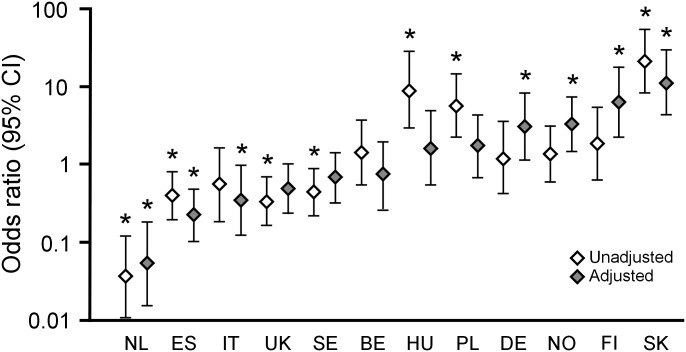
Unadjusted and adjusted odds ratios for FPs advice to take time off work for LRTI by network. Country as a predictor was evaluated with the clinical variables (presented in Table 4) and was included in the multivariable logistic regression with family practitioners fitted as random effect. *P<0.05.

Advice to take time off work was given to 55.6% employed patients, and the proportion varied from 7.6% in the Netherlands to 89.2% in Slovakia (Fig 1A). The majority (88.2%) were advised to take seven days or less off work. None of Finnish or Dutch patients were advised to take time for longer than 7 days, in contrast to 35.5% of Polish and 27.0% of Slovak patients (Table 2). Of the 145 (9.0% of employed participants) who reported obtaining sickness certification as main reason for consulting, 125 (86.2%) were advised to take time off work, with 114 (78.6%) being both advised to take time off work and received a formal sick certificate (Fig 1B). Fig 2 shows odds ratios (ORs) for advice to take time off work by network, unadjusted or adjusted for clinical presentation.

**Table 2:** Advice to take time off work according to patients’ characteristics by country of residence (network). BE—Belgium, DE—Germany, ES—Spain, FI—Finland, HU—Hungary, IT—Italy, NL—Netherlands, NO—Norway, PL—Poland, SE—Sweden, SK—Slovakia, UK—United Kingdom. Data were presented as mean±SD or median and interquartile range (Q1-Q3) or percentage related to the total number of cases in particular subgroups (%).

	BE (n = 48)	DE (n = 82)	ES (n = 74)	FI (n = 37)	HU (n = 168)	IT (n = 43)	NL (n = 6)	NO (n = 59)	PL (n = 93)	SE (n = 49)	SK (n = 174)	UK (n = 66)
Age (years)	41.2±10.5	39.3±12.6	39.2±12.0	42.2±10.7	38.7±10.5	42.8±9.9	44.8±2.6	43.2±10.5	40.1±10.0	46.7±9.2	39.3±10.8	45.0±11.3
Males (%)	58.1	85.7	40.9	70.0	82.2	35.1	9.7	57.1	66.7	25.5	80.8	32.1
Females (%)	53.5	55.9	35.9	62.5	78.3	43.5	6.3	54.9	79.7	39.8	94.3	37.6
Part time work (%)	40.0	60.0	27.0	57.1	62.5	33.3	6.5	36.0	55.6	40.5	80.0	28.3
Full time work (%)	60.6	66.3	40.0	64.7	81.4	42.4	9.1	61.7	78.3	33.0	89.7	38.1
Office work (%)	52.0	62.7	39.0	63.2	77.4	43.4	6.8	57.1	77.0	33.3	87.3	37.0
Manual work (%)	61.1	65.2	36.1	64.1	80.9	33.3	10.0	54.7	72.0	36.3	92.8	32.9
Current smokers (%)	65.4	65.0	44.9	75.0	73.8	37.5	13.0	53.3	76.8	38.1	88.9	35.7
Feeling unwell before presentation (days)	3 (2–6)	4 (3–7)	3 (2–5)	4 (3–7)	2 (2–3)	3 (2–9)	6.5 (4.5–8.25)	5 (4–8)	4 (2–5)	7 (5–9)	3 (3–5)	4.5 (4–7)
Number of symptoms	9 (8–10.75)	8 (7–9)	8 (7–9)	9 (7–10)	8 (5–9)	7 (6–9)	9 (7.75–12.5)	9 (8–10)	9 (7–10)	10 (8–10.5	8 (6–10)	10 (8–11)
Symptom severity score (%)	36.9±11.6	37.2±11.9	28.7±12.2	37.7±11.4	27.7±13.8	27.7±11.8	44.8±18.3	38.0±10.9	37.0±13.2	42.1±12.3	29.2±12.3	42.5±13.7
Discoloured phlegm (%)	69.4	71.7	31.8	63.9	85.1	55.3	7.9	50.8	76.9	28.4	88.2	35.5
^1^Comorbidity (%)	62.5	64.3	39.3	66.7	84.6	47.1	13.0	61.9	64.7	30.4	93.1	34.1
Auscultation abnormality (%)	71.4	65.8	39.6	84.0	80.2	45.9	15.6	64.7	82.3	38.6	91.0	42.7
Diagnosis of URTI (%)	48.6	60.0	29.5	50.0	40.0	22.9	0	42.1	79.2	12.5	97.6	32.4
Diagnosis of LRTI (%)	66.7	69.5	43.3	68.3	86.1	52.1	12.1	61.1	79.6	39.4	88.2	32.9
Antibiotics prescribed (%)	77.8	73.8	42.6	66.7	84.8	43.0	12.9	65.5	81.6	42.3	91.9	37.4
Advised delay of antibiotic treatment (%)	75.0	100.0	20.0	100.0	81.0	46.2	0	33.3	80.0	20.0	100.0	40.5
Advised for more than 7 days (%)	2.1	3.7	1.4	0	4.2	11.6	0	5.1	35.5	8.2	27.0	3.0

^1^comorbidity (diabetes, or cardiovascular- or respiratory-related).

### Characteristics of patients advised to stay off work

In general, patients who were advised to take time off work more frequently had abnormal lung auscultation findings, had been sick for longer before presenting, reported higher number of symptoms at presentation, and their symptoms were more severe. They were also more frequently diagnosed with LRTIs and prescribed antibiotics (Table 3). The characteristics of the patients advised to take time off work in each country are summarized in Table 2.

**Table 3:** Characteristic of employed patients related to advice to take time off work by family practitioners. Data presented as mean±SD inflated for clustering or median (25%-75% IQR) or percentage related to the total number of cases in particular subgroups (%);

	Advised off work (n = 899)	Not advised off work (n = 717)	Significance
Age (years)	40.7±11.0	44.1±12.8	*P*<0.001
Males (%)	36.2	37.0	*P* = 0.738
Females (%)	63.8	63.0	
Full time work (%)	86.0	70.9	*P*<0.001
Office work (%)	49.3	53.4	*P* = 0.098
Manual work (%)	50.7	46.6	
Feeling unwell before presentation (days)	4 (2–6)	6 (3.5–10.0)	*P*<0.001
Auscultation abnormality (%)	58.8	37.1	*P*<0.001
Number of symptoms	8 (7–10)	7 (5–9)	*P*<0.001
Symptom severity score (%)	33.5±13.7	27.7±13.9	*P*<0.001
^c^Comorbidity (%)	14.3	17.0	*P* = 0.141
^d^Cough (%)	87.4	83.4	*P* = 0.02
^d^Phlegm production (%)	42.4	39.2	*P* = 0.195
Discoloured phlegm (%)	44.7	45.3	*P* = 0.806
^d^Shortness of breath (%)	25.5	23.6	*P* = 0.378
^d^Wheeze (%)	13.7	11.4	*P* = 0.178
^d^Coryza (%)	40.2	31.9	*P*<0.001
^d^Fever (%)	36.0	12.4	*P*<0.001
^d^Chest pain (%)	22.4	16.9	*P* = 0.006
^d^Muscleache (%)	28.8	18.5	*P*<0.001
^d^Headache (%)	36.2	25.5	*P*<0.001
^d^Disturbed sleep (%)	38.4	41.1	*P* = 0.258
^d^Feeling unwell (%)	57.8	40.6	*P*<0.001
^d^Interference in normal activities (%)	52.3	33.8	*P*<0.001
^d^Confusion (%)	1.2	0.7	*P* = 0.288
^d^Diarrhoea (%)	1.6	1.1	*P* = 0.447
Current smokers (%)	29.3	26.9	*P* = 0.300
Diagnosis of URTI (%)	23.4	32.4	*P*<0.001
Diagnosis of LRTI (%)	56.8	43.4	*P*<0.001
Antibiotic treatment (%)	63.6	41.0	*P*<0.001
^e^Advised delay of antibiotic treatment (%)	11.5	17.0	*P* = 0.025

^c^comorbidity (diabetes, or cardiovascular- or respiratory-related)

^d^reported as moderate or severe problem

^e^related to number of patients prescribed with antibiotics.

### Geographic variations

Since work absenteeism has been found to be lower in Southern European countries compared to Central and Northern Europe [21], we included geographical location as predictor of FPs’ advice to take time in our logistic regression. The ORs adjusted for variables presented in Table 4, were: 0.19 (95%CI: 0.10 to 0.36), *P*<0.001 for Southern Europe region (ES, IT, n = 303); 1.20 (95%CI: 0.75 to 1.92), *P* = 0.435 for Central Europe region (BE, DE, HU, NL, PL, SL, UK, n = 1009) and 3.13 (95%CI: 1.64 to 5.95), *P* = 0.001 for Northern Europe region (FI, NO, SE, n = 304).

**Table 4:** Predictors of advising off work among working patients with LRTI. Odds ratios (ORs) were calculated based on multilevel mixed-effects binomial logistic regression with family practitioners (FPs) and countries fitted as random effects (FPs nested within countries).

Predictor	Adjusted ORs (95%CI)	Significance
Age (decades)	0.85 (0.75 to 0.97)	*P* = 0.013
Male gender	0.99 (0.74 to 1.33)	*P* = 0.936
Current smoker	1.00 (0.73 to 1.37)	*P* = 0.991
Felling unwell before seeing FPs more than three days	0.47 (0.34 to 0.64)	*P*<0.001
Fever during illness	2.51 (1.84 to 3.41)	*P*<0.001
Feeling generally unwell	2.19 (1.42 to 3.36)	*P*<0.001
Interference in normal activities	4.49 (3.06 to 6.59)	*P*<0.001
Respiratory comorbidities	0.88 (0.56 to 1.38)	*P* = 0.576
Heart related comorbidities	1.44 (0.62 to 3.32)	*P* = 0.393
Diabetes	1.30 (0.53 to 3.18)	*P* = 0.571
Auscultation abnormalities	1.48 (1.03 to 2.13)	*P* = 0.033
Discoloured phlegm	0.86 (0.63 to 1.16)	*P* = 0.322
Antibiotics prescribed	1.49 (1.04 to 2.15)	*P* = 0.030
Diagnosis of LRTI	1.53 (1.08 to 2.16)	*P* = 0.015
Follow up arrangement	1.44 (0.99 to 2.07)	*P* = 0.054
^g^Sicknote certification needed (no self-certification)	3.43 (0.95 to 12.36)	*P* = 0.059

^g^In following countries: Poland (PL), Belgium (BE), Hungary (HU), Spain (ES), Slovakia (SK), Italy (IT)

### Predictors of advising off work

Multilevel mixed-effects binomial logistic regression, with FPs nested within countries (adjusted for age (decades), gender, follow up arrangement, antibiotics prescribed, felling unwell before seeing FPs more than three days, auscultation abnormalities, fever during illness, feeling generally unwell, interference in normal activities, diagnosis of LRTIs, self-sickness certification) showed that FPs advice to patients with LRTIs to take time off work within a network was associated with antibiotics prescription, auscultation abnormalities discovered and increased number of symptoms at presentation, and was most strongly associated with patients reporting interference in normal activities (Table 4). Residual intraclass correlations for the country and FPs variables were 0.253 and 0.245 respectively.

Predictors of FPs’ advice to take time off work among working patients with acute cough by network were presented in Table 5.

**Table 5:** Predictors of advising off work among working patients with LRTI by country of residence (network). BE—Belgium, DE—Germany, ES—Spain, FI—Finland, HU—Hungary, IT—Italy, NL—Netherlands, NO—Norway, PL—Poland, SE—Sweden, SK—Slovakia, UK—United Kingdom. Table presents the odds ratios with 95% CI based on results of multilevel mixed-effects binomial logistic regression with family practitioners fitted as random effect.

	BE (n = 86)	DE (n = 128)	ES (n = 197)	^2^FI (n = 58)	HU (n = 210)	IT (n = 106)	NL (n = 79)	NO (n = 106)	PL (n = 124)	SE (n = 140)	SK (n = 195)	UK (n = 187)
Age (decades)	1.01 (0.5–2.0) *P* = 0.980	0.88 (0.6–1.3) *P* = 0.473	0.71 (0.5–1.0) *P* = 0.036	1.22 (0.7–2.3) *P* = 0.529	1.18 (0.8–1.8) *P* = 0.421	0.80 (0.6–1.1) *P* = 0.218	0.79 (0.3–2.0) *P* = 0.622	0.50 (0.3–0.8) *P* = 0.006	0.97 (0.6–1.5) *P* = 0.889	0.86 (0.6–1.3) *P* = 0.452	0.70 (0.3–1.5) *P* = 0.347	0.95 (0.7–1.3) *P* = 0.731
Male gender	0.92 (0.3–3.2) *P* = 0.896	6.30 (1.7–23.0) *P* = 0.005	1.29 (0.6–2.8) *P* = 0.525	0.82 (0.1–5.1) *P* = 0.836	1.72 (0.7–4.1) *P* = 0.216	0.63 (0.3–1.6) *P* = 0.333	2.67 (0.4–19.8) *P* = 0.336	0.80 (0.3–2.3) *P* = 0.679	0.54 (0.2–1.5) *P* = 0.240	0.42 (0.2–1.1) *P* = 0.073	0.07 (0–0.5) *P* = 0.007	0.77 (0.4–1.5) *P* = 0.453
Felling unwell before seeing FPs more than 3 days	0.24 (0.1–1.0) *P* = 0.049	0.48 (0.2–1.4) *P* = 0.188	0.19 (0.1–0.4) *P*<0.001	0.17 (0–1.0) *P* = 0.054	0.29 (0.1–0.7) *P* = 0.008	0.51 (0.2–1.3) *P* = 0.148	0.80 (0.7–9.3) *P* = 0.856	0.30 (0.1–1.4) *P* = 0.121	0.23 (0.1–0.7) *P* = 0.013	0.41 (0.1–1.7) *P* = 0.213	0.21 (0–1.0) *P* = 0.054	1.06 (0.5–2.3) *P* = 0.882
Auscultation abnormalities	4.93 (1.0–24.9) *P* = 0.053	1.06 (0.3–3.3) *P* = 0.917	1.32 (0.5–3.5) *P* = 0.576	8.60 (1.8–40.3) *P* = 0.006	1.09 (0.1–8.9) *P* = 0.935	1.37 (0.5–3.7) *P* = 0.528	8.26 (0.6–108.2) *P* = 0.108	4.04 (1.2–13.3) *P* = 0.021	2.17 (0.7–7.0) *P* = 0.199	1.25 (0.5–3.3) *P* = 0.650	1.00 (0.2–5.2) *P* = 0.997	1.68 (0.8–3.7) *P* = 0.188
Discoloured phlegm	2.78 (0.8–9.5) *P* = 0.102	1.99 (0.7–5.6) *P* = 0.195	0.40 (0.2–1.1) *P* = 0.067	-	1.47 (0.6–3.8) *P* = 0.425	2.55 (1.0–6.5) *P* = 0.049	0.48 (0.1–3.4) *P* = 0.466	0.50 (0.2–1.5) *P* = 0.221	0.72 (0.2–2.3) *P* = 0.582	0.35 (0.1–0.8) *P* = 0.019	1.74 (0.3–10.6) *P* = 0.546	0.91 (0.5–1.8) *P* = 0.795
Antibiotics prescribed	2.82 (0.5–17.4) *P* = 0.265	1.31 (0.4–4.4) *P* = 0.656	2.69 (0.9–8.1) *P* = 0.079	-	4.73 (1.7–12.9) *P* = 0.002	0.99 (0.3–3.0) *P* = 0.984	1.60 (0.2–13.4) *P* = 0.666	1.52 (0.5–5.0) *P* = 0.485	4.75 (1.3–17.5) *P* = 0.019	1.98 (0.8–5.0) *P* = 0.149	17.41 (1.9–159.8) *P* = 0.012	0.94 (0.4–2.2) *P* = 0.880

^2^Final model, presented only the significant association controlled for age (decades) and gender.

### Advice to take time off work and patient reported recovery

The median day of self-reported recovery was slightly lower for patients advised to take time off work comparing to those without the advice (9 (IQR: 7–14) vs 10 (IQR: 7–15) respectively, *P* = 0.015). However multilevel mixed effects binomial logistic regression (FPs nested within countries) revealed that advising to take time off work adjusted for age (decades), gender, follow up arrangement, antibiotics prescribed, felling unwell before seeing FPs more than three days, auscultation abnormalities, fever during illness, feeling generally unwell, interference in normal activities, diagnosis of LRTIs, self-sickness certification (by patients) was not associated with recovery (defined as feeling recovered within less than 10 days after presentation): OR: 1.11 (95%CI: 0.83 to 1.50), *P* = 0.569.

## Discussion

There is large variation in whether FPs advise their patients with LRTIs in Europe about taking time off work, as well as the recommended duration of time off work, which is not explained by differences in patient reported illness duration.

We found that consulting a FPs early on in the illness (which could be motivated by a country-specific requirement for obtain a sickness certificate from a FP from the first day taken off sick), as well as reporting more, and more severe symptoms at presentation, increased the likelihood of being advised to take time off work. The periods for which patients are entitled to self certify and sick pay policies vary across Europe and would have influenced our findings. Auscultation abnormalities, diagnosing LRTI, and prescribing an antibiotic may reflect greater concern among FPs about illness severity, and thus increase the chances of them advising patient to take time off work.

We found that advice to take time off work was not significantly associated with patient reported recovery. We are not aware of published data on the frequency and recommended duration of advice to take time off work for acute cough/LRTIs. However, predictors of sickness certification generally have been evaluated. One study showed that FPs’ sickness certification was influenced by their past experience, education, individual clinical reasoning, knowledge of the evidence base, personal beliefs, and time pressure [22]. A Swedish study found that FPs' were concerned about inadequate policy regarding sickness certification [23]. An analysis of sickness certification for cough in Poland and Norway found that differences could not be explained on strictly clinical grounds and concluded that important differences in sickness benefits were probably contributing to the result [24]. The right to stay off work in some countries without obtaining a sick certificate from a FP may increase the probability of giving advice off work in those with a longer “delay” in presentation.

At least 145 countries ensure provision for paid sick leave for short or long-term illnesses, with 127 countries allowing for a week or more of paid sick leave annually [25]. The European Union (EU) and other European countries established a legal right to at least 20 days of paid leave per year, which patients may use for sickness recovery. The EU’s Working Time Directive (1993) set a minimum paid-leave of four weeks or 20 days per year for all EU member countries [26], but several EU member countries make provision for substantially more paid leave, such as France, which mandates 30 days of paid annual leave and Finland and Sweden which mandate 25 days [27].

Study group inventory showed that the level of compensation for workers while off sick differs by country. For example it was 75% of a usual salary in Hungary, 80% in Belgium and Poland, and up to 100% in Finland and Sweden, starting from the second day of illness. These benefits start from the first day of illness certification in Poland and Belgium, and is paid by the employer for up to 30 days, followed by social insurance for up to 180 days. In Belgium benefits last for up to 28 days and only 60% of salary is compensated by social insurance. In Spain these benefits are paid by social insurance, but only once more than a limit of 15 days of illness per year has been reached.

A US survey found employed adults had continued to attend work while ill because of financial concerns [28]. Another US survey found that many workers who were eligible for paid time off sick, were nevertheless financially penalized for taking time off work and thus continued to work when unwell; 11% reported they had lost a job because of taking time off for illness, and 11% reposted they or a family member have been “fired, suspended or otherwise penalized for taking time off for illness” [29]. More detailed analysis of the role of “policy” factors requires further research and lies beyond of the scope of this study.

A strength of our study is that it was based on FPs recording their actual decisions and involved a large patient sample in a wide range of contrasting counties. Weaknesses included self-reported of data and possible practice selection bias: for example, only teaching practices with FP trainees recruited patients in Poland. Time constraints during consultation might have influenced the provision of advice about taking time off work. Analysis did not include cultural factors or varying country-level legal requirements. The results document wide variation in practice, but do not identify practice that is most beneficial for the individual patients, their family, and society.

## Conclusions

There is a large variation in FPs provision of advice to patients with cough/LRTI about taking time off work in Europe, which was not associated with patients’ reported recovery. There is a need to develop guidance that will promote consistent, evidence based advice from FPs to their patients about taking time off work for this common condition.
